# *Pimenta* Oil as a Potential Treatment for *Acinetobacter baumannii* Wound Infection: In Vitro and In Vivo Bioassays in Relation to Its Chemical Composition

**DOI:** 10.3390/antibiotics9100679

**Published:** 2020-10-07

**Authors:** Maha M. Ismail, Reham Samir, Fatema R. Saber, Shaimaa R. Ahmed, Mohamed A. Farag

**Affiliations:** 1 Microbiology and Immunology Department, Faculty of Pharmacy, Cairo University, Kasr El-Aini Street, Cairo 11562, Egypt; Reham.samer@pharma.cu.edu.eg; 2 Department of Pharmacognosy, Faculty of Pharmacy, Cairo University, Kasr el-Aini Street, Cairo 11562, Egypt; shaimaa.ahmed@pharma.cu.edu.eg (S.R.A.); mohamed.farag@pharma.cu.edu.eg (M.A.F.); 3 Department of Pharmacognosy, College of Pharmacy, Jouf University, Sakaka 2014, Saudi Arabia; 4 Department of Chemistry, School of Sciences & Engineering, The American University in Cairo, New Cairo 11835, Egypt

**Keywords:** *Acinetobacter baumannii*, MDR, biofilm, antimicrobial, *Pimenta*, *Myrtaceae*, wound infection, eugenol, 1,8-cineole, GC/MS

## Abstract

Bacterial biofilm contributes to antibiotic resistance. Developing antibiofilm agents, more favored from natural origin, is a potential method for treatment of highly virulent multidrug resistant (MDR) bacterial strains; The potential of *Pimenta dioica* and *Pimenta racemosa* essential oils (E.Os) antibacterial and antibiofilm activities in relation to their chemical composition, in addition to their ability to treat *Acinetobacter baumannii* wound infection in mice model were investigated; *P. dioica* leaf E.O at 0.05 µg·mL^−1^ efficiently inhibited and eradicated biofilm formed by *A. baumannii* by 85% and 34%, respectively. Both *P. diocia* and *P. racemosa* leaf E.Os showed a bactericidal action against *A. baumanii* within 6h at 2.08 µg·mL^−1^. In addition, a significant reduction of *A. baumannii* microbial load in mice wound infection model was found. Furthermore, gas chromatography mass spectrometry analysis revealed qualitative and quantitative differences among *P. racemosa* and *P. dioica* leaf and berry E.Os. Monoterpene hydrocarbons, oxygenated monoterpenes, and phenolics were the major detected classes. β-Myrcene, limonene, 1,8-cineole, and eugenol were the most abundant volatiles. While, sesquiterpenes were found as minor components in *Pimenta* berries E.O; Our finding suggests the potential antimicrobial activity of *Pimenta* leaf E.O against MDR *A. baumannii* wound infections and their underlying mechanism and to be further tested clinically as treatment for MDR *A. baumannii* infections.

## 1. Introduction

Wound healing is a complex biological process in the human body, achieved through programmed phases. Potential factors, including infection, can interfere with these phases and impair healing. There is increasing evidence that bacteria can delay healing processes by living within biofilm communities in which the bacteria are protected from the host immune system and to further develop antibiotic drug resistance. Referring to the previous findings, wound infection is detrimental to wound healing and there is a great demand for the development of more effective treatment for infected and poorly healing wounds [1,2,3].

*Acinetobacter baumannii* is one of the most important opportunistic nosocomial pathogens. This pathogen was recorded, with high incidence, among immune-compromised patients as the causative agent of wound infections [2]. Based on its pathogenicity, including biofilm formation and antimicrobial resistance, *A. baumannii* is regarded as a multidrug-resistant pathogen (MDR) that usually complicates wound infection prognosis [4], the phenomenon that warrants further investigation of both nosocomial and community-acquired infections [5]. The World Health Organization recognizes antimicrobial resistance as one of the three most important issues facing human health [6]. The need to develop new spectrum antibiotics to tackle multidrug resistance is still ongoing. Plant-based antimicrobials present a potential source of medication that is successful in treating infectious diseases while minimizing many of the side effects that are frequently associated with synthetic antimicrobials [7,8].

*Pimenta* genus is an important myrtaceous taxa that encompasses 15 species, mostly found in the Americas Caribbean area, and commonly used for several medicinal purposes [9,10]. *Pimenta dioica* and *Pimenta racemosa* are the most recognized in that genus to exhibit potential pharmacological effects owing to its rich essential oil composition [11]. Traditionally, different *Pimenta* plant parts have been utilized for the alleviation of common cold, viral infections, bronchitis, dental and muscle aches, rheumatic pains, and arthritis. Furthermore, its berry oil is reported to exhibit antimicrobial, antiseptic, anesthetic, and analgesic properties [12]. Essential oils of *Pimenta* are ethno-pharmacologically relevant in traditional management of microbial infections [5,13]. *Pimenta* spp. antimicrobial and antiviral activities have been previously reported against several microbes including *Pseudomonas aeruginosa, Escherichia coli, Staphylococcus aureus*, and antifungal activity against *Candida albicans, Aspergillus niger,* and *Abisidia corymbifera*. *P. racemosa* is used in folk medicine for the treatment of influenza, pneumonia, fever rheumatism, toothache, and abdominal pains [14,15,16]. 

Studies on *P. dioica* leaf essential oil showed that eugenol, methyl eugenol, β-caryophyllene, and myrcene were the most abundant constituents, while limonene, 1,8-cineole, terpinolene, β-caryophyllene, β-selinene, and methyl eugenol were the major constituents in other studies [13,17,18].

In the preset study, antimicrobial and antibiofilm activities of *Pimenta* essential oils (E.Os) grown in Egypt were evaluated against *A. baumannii* using in vitro and in vivo models to assess their role as antimicrobial agents in wound infections. To the best of our knowledge, this is the first study reporting on the in vitro and in vivo antimicrobial efficacy of *Pimenta* oils against *A. baumannii* pathogen.

## 2. Results and Discussion

A major goal of this study was to assess the potential antimicrobial effect for *Pimenta* essential oils (E.Os) represented by its different organ or species type. The minimum inhibitory concentration (MIC) values of the E.Os obtained from *Pimenta racemosa* and *Pimenta dioica* leaves and berries against 15 bacterial isolates were in the range of 0.51–5.2 µg·mL^−1^ (Table 1). Among the tested plant parts, leaves’ E.Os demonstrated the strongest antimicrobial activities in terms of smaller MIC values and were thus chosen for further in vivo wound infection. Eugenol exhibited the strongest antimicrobial activity with the lowest MIC values (ranging from 0.1 to 5.2 µg·mL^−1^ as compared to all tested E.Os). While *P. dioica* berry oil displayed the weakest antimicrobial activity (average MIC value 3.38 µg·mL^−1^) compared to other E.Os. Besides, MIC values of *β*-myrcene, a monoterpene hydrocarbon was higher than 4 µg.mL^−1^ against all tested strains. 

Although, the order of activity according to the average MIC values was as follows: 

Eugenol > *P. dioica* leaf/*P. racemosa* leaf > *P. racemosa* berry > cefepime > *P. dioica* berry.

No statistically significant difference was observed among the activities of the four tested E.Os, eugenol, while a statistically significant difference existed between cefepime and each of the tested E.Os and eugenol (Kruskal–Wallis test, Dunn’s multiple comparisons test, *p*-value < 0.0001).

Semiquantitative determination of biofilm formation by the examined five *A. baumannii* strains revealed that AB-R strain is a strong biofilm forming (BF), and both AB-8 and AB-16 are moderate BF while AB-11 and AB-13 strains are negative BF. Therefore, the effect of E.Os on biofilm formation and eradication was tested on three strains; AB-R, AB-8, and AB-16. 

**Figure 1:**
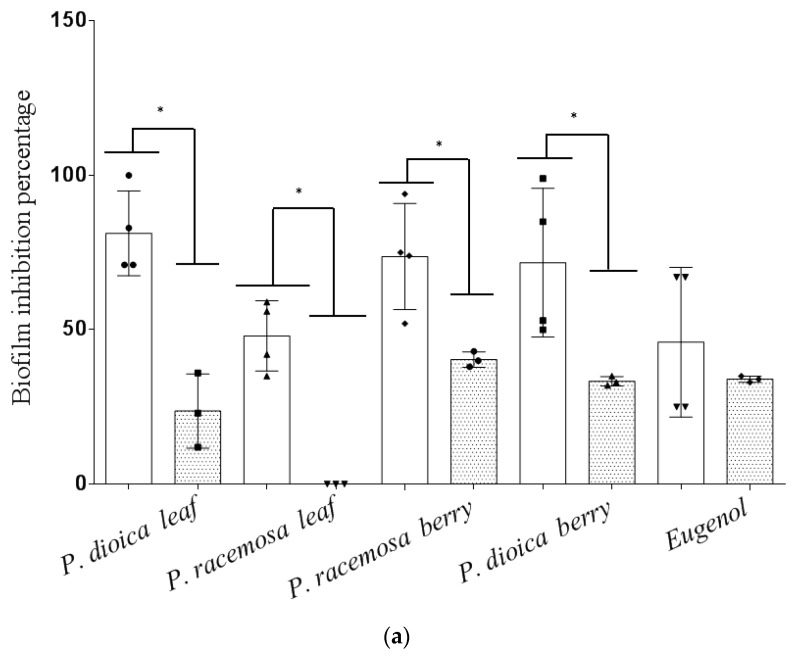

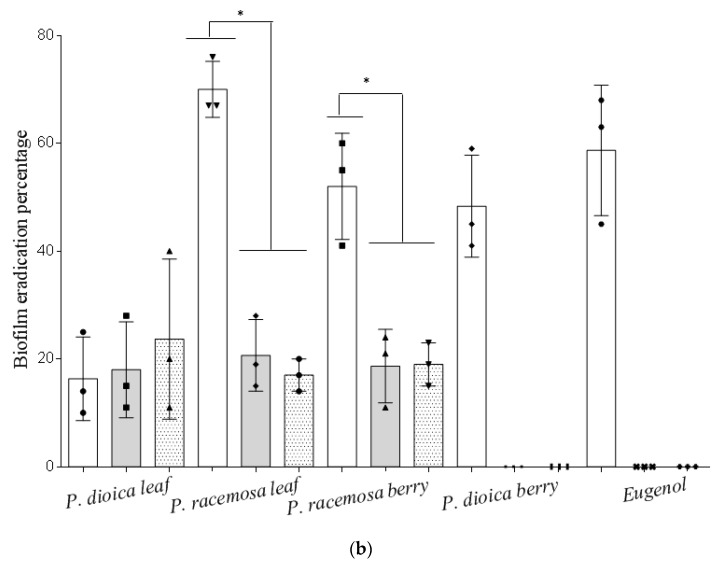
(**a**) Biofilm inhibition percentages of *Pimenta* essential oils/eugenol at a dose of 0.1× MIC against MDR AB-8 (white) and AB-16 (light shade) isolates. (**b**) Biofilm eradication percentages of the tested E.Os/eugenol at a dose of 0.1× MIC against MDR AB-8 (white), AB-R (gray), and AB-16 (light shade) isolates. The symbols in each column (●, ■, ▲, ◆, ▼, ×, +) represent each data point of the triplicate experiments performed for each E.O and eugenol against the tested bacterial isolates. Data represent the means of biofilm inhibition/eradication percentages ± SD, *n* = 3 (one-way ANOVA, followed by Tukey’s multiple comparisons, *p*-value < 0.0001). (*) Means statistically significant difference exists between columns.

Unexpectedly, no inhibition activity was detected against the strong biofilm formed by the standard *A. baumanii* strain (AB-R). However, all *Pimenta* oils, as well as eugenol, could inhibit biofilm formation by AB-8 and AB-16, at 0.1× MIC (0.05 and 0.10 µg·mL^−1^, respectively), except for *P. racemosa* leaf E.O, which could inhibit biofilm formed by AB-8 only at 0.05 µg·mL^−1^ (0.1× MIC). In detail, *P. dioica* leaf oil was found superior as antibiofilm relative to eugenol and other tested oils (Figure 1a). Comparing leaf to berry oil, *Pimenta* berry oils showed stronger effect as antibiofilm than that of *P. racemosa* leaf oil. The observed antibiofilm activity of *Pimenta* oils was higher than that of standard eugenol, though with no significant difference (one-way ANOVA, followed by Tukey’s multiple comparisons *p*-value < 0.0001). 

The same concentrations of all tested E.Os and eugenol, which inhibited biofilm formation, could also eradicate biofilm formed by AB-8, while only *P. dioica* leaf only, *P. racemosa* leaf and berry E.Os could eradicate biofilm formed by AB-R and AB-16 strains. Eradication of AB-8 strain biofilm by the E.Os and eugenol was more statistically significant than the eradication effect on biofilm formed by other strains (AB-R and AB-16), except for *P. dioica* leaf E.O (one-way ANOVA followed by Tukey’s multiple comparisons *p*-value < 0.0001) (Figure 1b). The reported biofilm eradication activity of *Pimenta* E.Os herein is in agreement with Vázquez-Sánchez et al. [19] who demonstrated the eradication of 24h-old biofilm of *L. monocytogenes* by *Pimenta pseudochariophyllus* E.O at doses of 2.75% and 3% within 30 min of application. In another study, the same E.O could also eradicate 24h-old *Staphylococcus aureus* biofilm at doses of 1.75% and 3%. Eugenol and 1,8-cineol were among the main reported components of this E.O [20].

In this study, eugenol could eradicate the biofilm formed by AB-8 strain only and did not affect AB-R or AB-16 preformed biofilms. In a previous study performed by Yadav et al. [21], it was reported that eugenol (at 0.5× MIC) could eradicate MRSA and MSSA preformed biofilms, these authors reported that within 6 h, eugenol could reduce biomass and cell viability of the preformed biofilms by two mechanisms, the first is by lysis of bacterial cells within biofilms, and the second mechanism is via disruption of the cell-to-cell connections leading to biofilm organization disassembly.

The antimicrobial and antibiofilm activity of *Pimenta* spp. E.Os presented herein, is in agreement with recent studies reporting the potential of myrtaceous plants as efficient inhibitors of bacterial quorum sensing and to possess pronounced antimicrobial and antibiofilm activities [18,22]. A study performed by Vasavi et al. [23] revealed a strong antiquorum sensing activity for the ethyl acetate fraction of ethanolic extract of *P. dioica,* this fraction could inhibit AHL-mediated violacein pigment production in *Chromobacterium violaceum,* ATCC12472, at dose-dependent manner, the authors related this effect to polyphenolic compounds like the E.O components of the tested extract.

The minimum bactericidal concentration (MBC) values of *Pimenta* oils were estimated at different dose levels, where all of the tested oils exhibited bactericidal effect after 24 h incubation. *P. dioica* leaf oil exhibited the most pronounced bactericidal action at 1 µg·mL^−1^ (2× MIC) against the tested AB-8 isolate (Table 2). Both *P. racemosa* leaf and berry E.Os exhibited the same bactericidal activity at 2.08 and 2.76 µg·mL^−1^, respectively. The bactericidal activity of eugenol was inferior to that of *P. dioica* leaf oil. In addition, *P. dioica* berries E.O at 41.4 µg·mL^−1^ (8× MIC) results in two-log cycle colony count reduction as compared to the control untreated bacteria. While, as a consequence, higher concentration, than 41.4 µg·mL^−1^, of this E.O was required to achieve a complete bactericidal activity which was not tested. 

**Figure 2:**
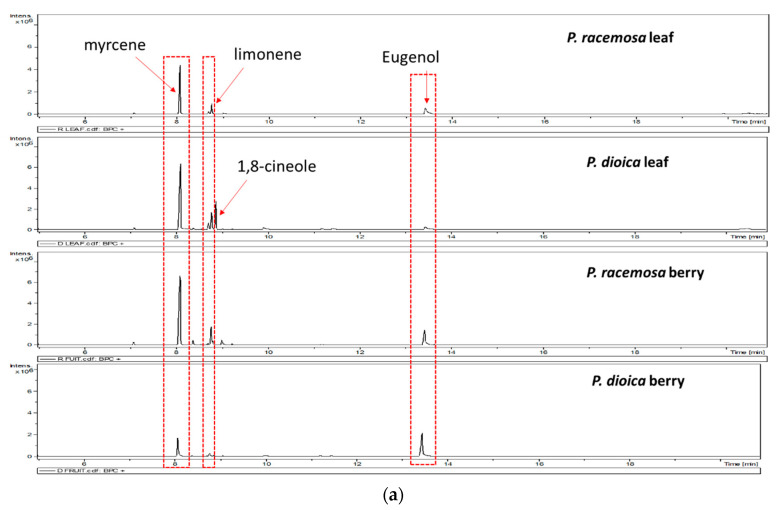

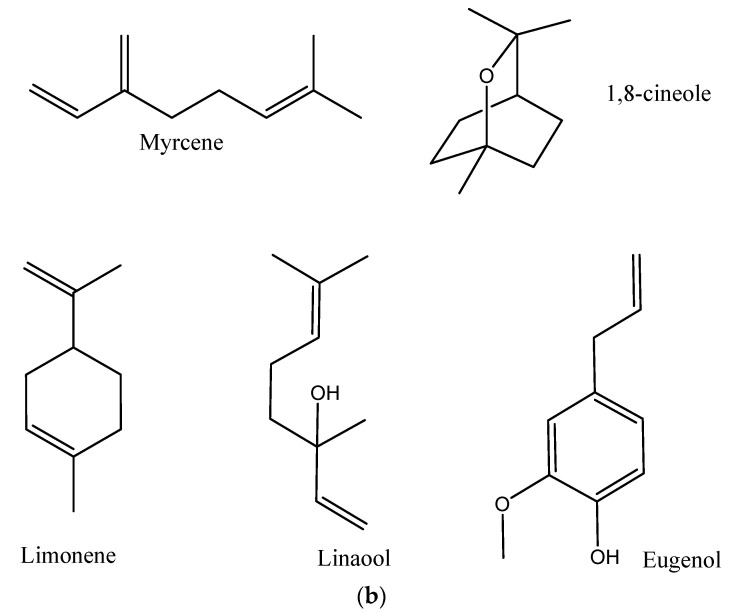
(**a**) Representative GC/MS chromatograms of *P. racemosa* and *P. dioica* leaf and berry essential oils. (**b**) Structures of the major volatile constituents detected in *P. racemosa* and *P. dioica* essential oils.

GC/MS analysis was employed to account for volatiles’ compositions in tested *Pimenta* E.Os and mediating for the difference observed in the antimicrobial effects, results are depicted in Table 3. It was revealed that monoterpene hydrocarbons prevail in both *P. racemosa* and *P. dioica* leaf oils, constituting up to 64.4% predominance, with *β*-myrcene as the major identified component accounting for 39.6% and 44.1%, respectively (Figure 2a,b, Table 3).

Next, limonene was identified as a second major monoterpene hydrocarbon in *P*. *racemosa* leaf and berry at 14–15%, and to less extent in *P. dioica* berry oil (4.6%). In contrast, 1,8-cineole recorded the highest levels in *P. dioica* leaf oil (18.8%) compared to its berries, this was previously reported in the Cuban oil of *P. dioica* leaf (14.69%) [24]. 1,8-Cineole, among reported volatiles, is likely to mediate for the observed antimicrobial effects, in agreement with previously reported its potent antimicrobial effect against *Streptococcus mutans* [25]. In a recent study, Merghni et al. [26] recorded high MIC values displayed by 1,8-cineole against MRSA strains (≥29 mm) and suggestive for its positive effect against MDR bacteria. The antibiofilm activity of *P.dioica* leaf oil could be correlated to its enrichment in 1,8-cineole (18.80%). A study performed by Santos and Rao (2001) [27] reported the ability of 1,8-cineole to alleviate gastric mucosa injury and damage induced by absolute ethanol in rats. Stojkovic et al. [28] investigated the inhibitory effect of 1,8-cineole on apple rot disease caused by *Aspergillus niger* at concentrations of 3% and 6% of 1,8-cineole applied for 3 days, 100% inhibition of apple rot disease was observed. Merghni et al. [26] also reported 1,8-cineole biofilm inhibition effect against *S. aureus* ATCC 6538. Our results suggest possible synergistic antibiofilm activities of volatile oil constituents in *Pimenta* spp.

Eugenol dominated *P. dioica* berries essential oil composition (65.6%). Eugenol was found as a second predominant constituent in *P*. *racemosa* leaf and berry E.Os at comparable level of 27–31%. In the present study, it was observed that eugenol could inhibit *A. baumannii* biofilm formation at 0.02 µg·mL^−1^ which is in agreement with a study conducted by Kim et al. [29]. Where, more than 75% inhibition of *E. coli* O157:H7 biofilm was observed by eugenol, *P. racemose*, and *P. dioica* berries oils at slightly higher levels (0.052 µg·mL^−1^), though a different organism was tested. Noteworthy, eugenol is well-characterized as an antimicrobial agent against oral pathogens, infective diseases, and foodborne infections [30,31]. 

Further, sesquiterpene hydrocarbons were only detected in *Pimenta* berries oils at low levels, reaching 0.3% and 1.4% in *P. racemosa* and *P. dioica* berries, respectively. Major sesquiterpenes included *δ*-Cadinene in *P. dioica* berry oil followed by *β*-caryophyllene, *α*-humulene, germacrene D, and *α*-cubebene. 

Although cefepime is considered to be the drug of choice for treatment of *A. baumanii* infections [32], its MIC values varied between 0.1 and 10 µg·mL^−1^ which can be attributed to the variable susceptibilities of the MDR isolates used herein. 

**Figure 3:**
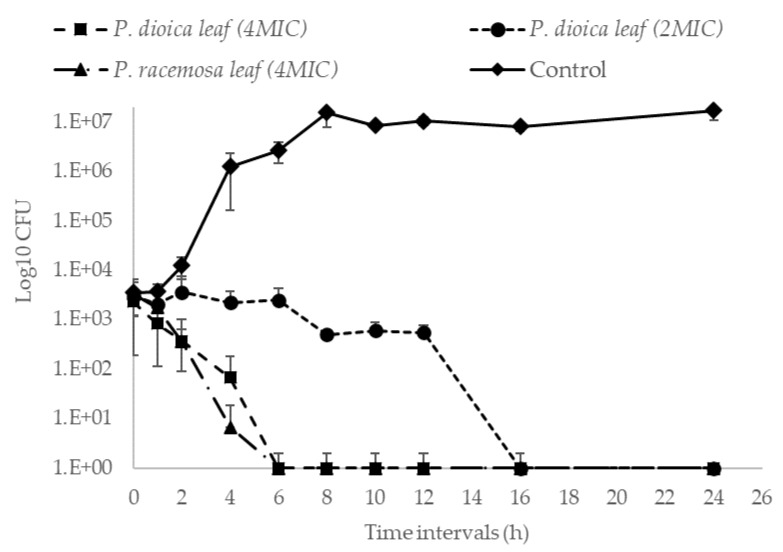
The killing kinetics of *Pimenta dioica* and *Pimenta racemosa* leaf essential oils tested against AB-8 isolate. (■) *P. dioica* leaf E.O. tested at concentration of 2.0 µg·mL^−1^ (4× MIC), (●) *P. dioica* leaf E.O. tested at concentration of 1.0 µg·mL^−1^ (2× MIC), (▲) *P. racemosa* leaf E.O. tested at concentration of 2.0 µg·mL^−1^ (4× MIC). Data is represented by means of the number of recovered colonies counted at each time point ± SD, *n* = 3.

Accordingly, killing kinetics of AB-8 strain by *P. dioica* and *P. racemosa* leaf oils were further investigated. Within 6h of incubation, both oils could kill AB-8 strain at 2 µg·mL^−1^ (4× MIC) (Figure 3), while longer time (16h) was needed for *P. dioica* leaf oil to kill the bacterium at 1 µg·mL^−1^ (2× MIC). In contrast, the E.O of *P. racemosa* leaf, at 1 µg·mL^−1^, could not kill the bacteria nor achieve any level of bacterial colony count reduction.

The observed bactericidal action of eugenol is attributed to several mechanisms, involving alteration of bacterial membrane permeability causing leakage of ions leading to cell death [33], and in correlation to its phenolic nature which facilitates its binding to target proteins on the bacterial cell [34]. On the other hand, the monoterpene hydrocarbons *viz*., *β*-myrcene, *α*-pinene, and limonene are generally known to exert less antibacterial effects in comparison to oxygenated monoterpenes and phenolics [35] and might act to synergize the main action of cineole and eugenol in *Pimenta* oil, thus accounting for the superior antibiofilm effect of the oil compared to eugenol standard tested alone (Figure 1). The lipophilic nature of terpene hydrocarbons greatly affects bacterial membrane permeability and might improve uptake of E.O components into bacterial cells enhancing its penetration into the bacterial biofilms and thus exerting its antimicrobial and antibiofilm action and further potentiating the effect of other concomitant antibiotics [36,37]. 

According to the obtained MIC, MBC values, and antibiofilm activities, both *P. dioica* and *P. racemosa* leaf oils were selected to test their in vivo effectiveness as antimicrobial agents in a mouse model of wound infection as they showed the lowest MIC and MBC values together with strong antibiofilm activity (Table 1, Figure 1 and Figure 3).

*Acinetobacter* is regarded as one of the pathogens involved in nosocomial infections and outbreaks. In a previous epidemiological study, skin colonization with *Acinetobacter* spp. was found at a higher rate in hospitalized patients than in healthy people [38], thus a wound infection animal model was tested.

**Figure 4:**
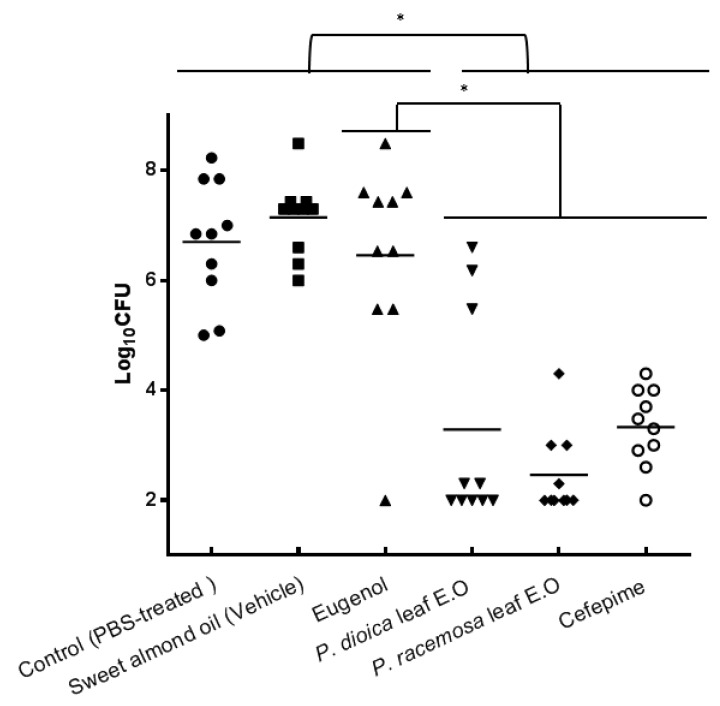
The antimicrobial effect of *Pimenta dioica* and *Pimenta racemosa* leaf essential oils and eugenol at 5.2 µg·mL^−1^ (10× MIC) concentration and cefepime at 25 µg·g^−1^ of mouse weight, tested against AB-8 isolate in a skin wound infection mouse model. Data is represented by the mean colony counts recovered from each of the six infected groups ± SD, six days post-infection. (●) data points of control infected PBS-treated group, (■) data points of sweet almond-treated group (negative (vehicle) control group), (▲) data points of eugenol-treated group, (▼) data points of *P. dioica* leaf E.O-treated group, (♦) data points of *P. racemosa* leaf E.O-treated group, (○) data points of cefepime-treated group (positive drug control group). *n* = 10 mice per group. (One-way ANOVA followed by Tukey’s multiple comparisons test, *p*-value = 0.0047). (*) Means statistically significant difference exists between groups.

Interestingly, the microbial load of infected wounds in the mice groups treated with each of the E.Os and cefepime demonstrated a statistically significant reduction compared to other groups including the group treated by eugenol (one-way ANOVA followed by Tukey’s multiple comparisons test, *p*-value = 0.0047). This is suggestive that the E.O is more active than standard eugenol due to possible synergistic action of volatile oil constituents. Three log cycle reduction was observed in the total bacterial count of groups treated with E.Os compared to infected PBS-treated control group (Figure 4). *P. dioica* leaf oil did not show a significantly higher activity than that of *P. racemosa* on reducing bacterial count recovered from both treatments. No significant differences were recorded among the infected untreated group and groups treated with either almond oil vehicle or eugenol indicating that eugenol was less active in this in vivo assay. Nevertheless, Kim et al. [29] showed that eugenol could prolong the lifespan of a nematode worm infected with EHEC. Yadav et al. [21] reported eugenol, at sub-MIC, to be effective in reducing *S. aureus* colonization by 88% in a rat middle ear infection model.

The antimicrobial activity of *P. dioica* leaf oil could be attributed to its 1,8-cineole enriched content in addition to eugenol [39]. Cefepime showed a better reduction of microbial load than that observed due to *P. racemosa* leaf E.O and a comparable effect to *P. dioica* leaf E.O but these differences were statistically nonsignificant (one-way ANOVA followed by Tukey’s multiple comparisons test, *p*-value = 0.0047).

Another study performed by Karumathil et al. [40] revealed the in vitro ability of eugenol to reduce *A. baumannii* adhesion and invasion to human keratinocytes and also to inhibit biofilm formation in an in vitro collagen matrix wound model.

Nevertheless, few studies have investigated the in vivo antimicrobial effects of plant-derived E.Os against *A. baumannii* infections and likely to exert a stronger effect being composed of complex volatiles compared to a single chemical component. This study is the first to report the in vivo antimicrobial effect of *P. dioica* and *P. racemosa* E.Os in an *A. baumannii* wound infection model. In the same context, Tsai et al. [41] screened 30 Chinese herbs for antimicrobial effect against extensively drug-resistant *A. baumannii* isolates. Where, only *Scutellaria barbata* aqueous extract showed the highest activity in vitro and is likely to be mediated by polar type compounds in contrast to lipophilic nature of E.O reported herein and suggesting that different chemically based metabolites could evoke antimicrobial action against *A. baumannii*. 

## 3. Materials and Methods

### 3.1. Plant Material

Leaves and berries of *Pimenta dioica* (L.) Merr. and *Pimenta racemosa* (Mill.) J.W. Moore were collected from Zohria Botanical Garden in May 2017. They were identified by Mrs. Therese Labib, Consultant of Plant Taxonomy at the Ministry of Agriculture and Orman Botanical Garden, Giza, Egypt. A voucher specimen of the plant material was deposited at Pharmacognosy Department, Faculty of Pharmacy, Cairo University, Egypt.

### 3.2. Preparation of Essential Oil

Leaf and berry of *Pimenta* species under investigation were hydro-distilled separately in a Clevenger-type apparatus for 4h, according to the procedure described in the Egyptian pharmacopeia (2005) [42]. The obtained oils were dehydrated by filtration through anhydrous sodium sulfate and kept in a refrigerator for subsequent GC/MS analysis and antimicrobial assays.

### 3.3. GC/MS Analysis of the Volatile Oil Composition

E.Os. prepared from *P. dioica* and *P. racemosa* leaf and berry were subjected to GC/MS analysis. The injection volume was 1 µL. The instrument was controlled by the Shimadzu Class-5000 Version 2.2 software containing a NIST62 (National Institute of Standards and Technology) MS library. Volatile components were separated on a DB5-MS column (30 m length, 0.25 mm inner diameter, and 0.25 µm film thickness (J&W Scientific, Santa Clara, CA, USA). Injections were made in the split mode for 30 s, and the gas chromatograph was operated under the following conditions: injector 220 °C and column oven 40 °C for 3 min, then programmed at a rate of 12 °C/min to 180 °C, kept at 180 °C for 5 min, and finally ramped at a rate of 40 °C/min to 220 °C and kept for 2 min, He carrier gas at 1 mL/min. The transfer line and ion-source temperatures were adjusted at 230 and 180 °C, respectively. The HP quadrupole mass spectrometer was operated in the electron ionization mode at 70 eV. The scan range was set at 40–500 *m/z*.

The percentages of different components in each oil sample were determined by computerized peak area measurements relative to each other. Volatile components were identified using the procedure described in Farag and Wessjohann [43]. The peaks were first deconvoluted using AMDIS software (www.amdis.net) and identified by its retention indices (RI) relative to n-alkanes (C6–C20), mass spectrum matching to NIST, WILEY library database. Results are depicted in Table 2.

### 3.4. Bacterial Isolates and Culture Conditions

Fourteen MDR clinical isolates of *Acinetobacter baumanii*, in addition to one reference strain (ATCC 19606), were used in this study. They were isolated from patients in Kasr El-Ainy hospital, Cairo, Egypt [44]. The strains were grown aerobically on Luria–Bertani (L.B) agar/broth (Oxoid, UK) with shaking at 180 rpm at 37 °C for 24 h. 

### 3.5. Determination of the Minimum Inhibitory Concentration (MIC) by Agar Microdilution Technique 

MIC was determined according to the method described by Golus et al. [45]. Briefly, freshly prepared two-fold serial dilutions of the test essential oil (E.O.)/standard volatiles (eugenol and myrcene) were prepared in dimethyl sulfoxide (DMSO). In total, 1 µL of each E.O dilution was dispensed into the U-shaped bottom 96-well microplates. Aliquots of 100 µL of the molten L.B agar medium (at ≈60 °C) were mixed with E.O. in each well before solidification. Then, 2 µL of a freshly prepared bacterial suspension (in sterile normal saline (10^7^ CFU·mL^−1^)) was inoculated onto the surface of the solidified mixture (L.B agar medium + E.O/standard compound).

The lowest concentration showing no visible bacterial growth after incubation at 30 °C for 24 h were recorded. Negative and positive controls (L.B agar medium + DMSO and L.B agar medium + DMSO + bacteria, respectively) and antibiotic control (cefepime) were included. The test was performed in triplicate.

### 3.6. Effect of Essential Oils on Biofilm Formation and Eradication

Five *A. baumannii* strains, namely AB-8, AB-R, AB-11, AB-13, and AB-16, were semiquantitatively examined for their biofilm forming ability in a 96-well flat bottom microtiter plate according to a formula reported by Naves et al. [46]:

BF=AB−CW

where:

BF, biofilm formation

AB, stained bacteria cells attached to the wells

CW, stained control wells

Bacterial isolate is considered a strong, moderate, weak, or negative biofilm forming if BF is ≥0.300, 0.200–0.299, 0.100–0.199, and <0.100, respectively [46].

Then, strong or moderate biofilm forming strains were selected to perform biofilm inhibition/eradication assays.

#### 3.6.1. Biofilm Inhibition Assay

The assay was performed according to Hussein et al. [47]. To estimate the ability of E.O/standard to inhibit biofilm formation by bacteria, an overnight culture of the corresponding isolate in L.B broth, adjusted at OD_600_ of 1, was diluted 1:100 in fresh LB broth. Then, 200 µL of each culture were dispensed in the wells of 96-well flat-shaped bottom microplates. A total of 1 µL of sub-MIC concentrations of each E.O./standard compound (0.5× MIC, 0.25× MIC, and 0.1× MIC) was mixed with LB broth in the wells and then incubated overnight at 37 °C. Growth was monitored by recording the optical density at 600 nm using microplate reader (BioTek Synergy 2, Winooski, VTUSA). Plates were then washed gently twice with phosphate-buffered saline to remove planktonic cells without disturbing the biofilm. Plates were left to completely dry in a laminar air flow cabinet. For biofilm visualization, 200 µL of 0.5% crystal violet solution were added to each well and left still for 30 min. Plates were then washed twice with distilled water to remove excess stain and left to dry completely. To extract the color of crystal violet, 150 µL of 99% ethanol were added per well and plates were left for 15 min with gentle shaking. The extracted color was measured colorimetrically at 570 nm in order to estimate the extent of biofilm formation. Negative and positive controls (broth only and broth inoculated with bacteria, respectively) were included. The test was performed in triplicate.

#### 3.6.2. Biofilm Eradication Assay

Test strains were allowed to form mature biofilm for 24 h in the wells of 96-well flat-shaped bottom microplates under the same conditioned mentioned in biofilm inhibition assay without any E.O or inhibitory compound. After that, the spent media containing the planktonic cells were discarded and fresh L.B broth with different concentrations of the test E.O./standard compound (0.5× MIC, 0.25× MIC, and 0.1× MIC) were added to the mature biofilm in the wells. The plates were further incubated for 24 h at 37 °C, then they were washed and stained for biofilm visualization according to the steps mentioned in biofilm inhibition assay. Negative and positive controls (broth only and broth inoculated with bacteria, respectively) were included. The test was performed in triplicate.

### 3.7. Determination of the Minimum Bactericidal Concentration (MBC) by Broth Microdilution Technique

Amounts of 100 µL of L.B broth were dispensed into the U-shaped bottom 96-well microplates, concentrations equivalent to 1× MIC, 2× MIC, 4× MIC, 6× MIC, and 8× MIC of each E.O/standard compound, against *A. baumannii* strain-8 (AB-8), were tested. In total, 2 µL of a freshly prepared bacterial suspension (10^7^ CFU·mL^−1^) was inoculated into the mixture, plates were then incubated at 37 °C for 24 h. Viable colony count on L.B agar medium was performed to determine the MBC at which no colonies appear. Negative and positive controls were included (L.B broth + DMSO and L.B broth + DMSO + bacteria, respectively). The test was performed in triplicate.

### 3.8. Kill Kinetics Assay

Time–kill kinetics of *P. dioica* and *P. racemosa* leaf E.O/standard compound against AB-8 strain was performed according to the method of [48]. The killing kinetics of the E.O were assayed at the bactericidal concentrations. First, 2 µL of 10^7^ CFU·mL^−1^ suspension of AB-8 strain was incubated with MBCs of E.Os in L.B broth for up to 24 h. Then, samples of each test were withdrawn at time intervals of 0, 1, 2, 4, 6, 8, 10, 12, 16, and 24 h, diluted and subjected to viable colony count on L.B agar medium. Plates were then incubated at 37 °C for 24 h and then the visible colonies were counted. Positive and negative controls of DMSO + AB-8 in L.B broth and DMSO in L.B broth, respectively, were included. The assay was performed in triplicate.

### 3.9. In Vivo Wound Infection Animal Model 

#### 3.9.1. Ethical Statement 

All experiments involving animals were conducted according to the ethical policies and procedures approved by the ethics committee of the Faculty of Pharmacy, Cairo University, Cairo, Egypt (Approval no. MI-2364).

#### 3.9.2. Experimental Design and Induction of Infection

The animal model was performed according to Wang et al. [49] as follows: 60 adult 6–8 weeks old male mice weighing 25–35 g were obtained from the Modern Veterinary Office for Laboratory Animals, Giza, Egypt. Animals were kept in cages under well-defined and standardized conditions (humidity and temperature-controlled room; 12-h light and 12-h dark cycle). The mice were initially examined to exclude any sign of skin inflammation and were fed with a standard dry food and water on demand.

Back hair was clipped from the cervical to mid-lumbar dorsum, and the skin was rinsed with ethanol. The skin on the shaved back of the mice was lifted with forceps and a 1.0 × 1.0 cm full thickness excisional wound was made by removing full thickness skin with a scissors. Infection was induced by adding aliquots of 10 µL freshly prepared suspension of isolate AB-8 in LB broth containing 10^7^–10^8^ CFU·mL^−1^ into the wound and allowed to be absorbed (the experiment was performed twice, once using 10^7^ and then using 10^8^ CFU·mL^−1^. Each time, the used inoculum was determined by viable colony count at time zero). *P. dioica* and *P. racemosa* leaf E.O/eugenol were prepared as 10× MIC which is equivalent to 5× MBC (5.2 µg·mL^−1^) in sweet almond oil as the vehicle. Cefepime was used as a positive drug control at a dose of 25 µg·g^−1^ of mice weight [31].

Twenty-four hours post-infection, animals were divided into six groups, 10 mice each, and treatment proceeded for 6 days. 

Each group received 25 µL of respective treatment:

- Group 1: *P. dioica* leaf E.O. dissolved in sweet almond oil (5.2 µg·mL^−1^).
- Group 2: *P. racemosa* leaf E.O. dissolved in sweet almond oil (5.2 µg·mL^−1^).
- Group 3: Eugenol dissolved in sweet almond oil (2 µg·mL^−1^)
- Group 4: Cefepime solution (25 µg per gram of mouse weight).
- Group 5: Vehicle (sweet almond oil).
- Group 6: Phosphate-buffered saline.

Animals were then sacrificed and the wounded skin was removed for viable count technique (counts recovered from groups were compared), in brief, the wounded skins were cut into small pieces using sterile scalpels and then homogenized with PBS. Then, 20 μL aliquots of each suspension were 10-fold serially diluted using PBS, then, 10 μL of each dilution was spotted onto the surface of L.B agar medium, incubated at 37 °C for 24 h and then, the recovered colonies were counted and groups compared. 

### 3.10. Statistical Analysis

One-way ANOVA, followed by Tukey’s multiple comparisons and Kruskal–Wallis test, Dunn’s multiple comparisons test (*p*-value < 0.05) were performed using GraphPad Prism 6.01 (GraphPad Software, Inc., San Diego, CA, USA).

## 4. Conclusions

Chemical characterization of *Pimenta racemosa* and *Pimenta dioica* leaf and berry E.Os revealed the abundance of oxygenated monoterpenes and phenolics’ which are likely to contribute to the oil antimicrobial effect. The strong antimicrobial and antibiofilm activities of both *P. dioica* and *P. racemosa* leaf oils pose them for future incorporation in treatment of MDR *A. baumanii* infections. Our future perspective will focus on designing and characterization of a suitable pharmaceutical nano-formulation containing the *Pimenta* E.Os, to be further up-scaled to be incorporated in antimicrobial and antibiofilm adjuvant therapy after sufficient clinical trials. 

## Figures and Tables

**Table 1:** The minimum inhibitory concentration values (MIC), in µg·mL^−1^, of tested *Pimenta* essential oils, eugenol, and cefepime antibiotic tested against the clinical isolates of *Acinetobacter baumanii* (AB), the results are represented as the mean of three replicates ± standard deviation.

Isolate	*P. dioica* Leaf ^a^	*P. racemosa* Leaf ^a^	*P. dioica* Berry ^a^	*P. racemosa* Berry ^a^	Eugenol ^a^	Cefepime ^b^
AB-1	0.69 ± 0.2	0.52 ± 0.0	0.69 ± 0.2	0.69 ± 0.2	0.10 ± 0.0	10 ± 0.0
AB-2	0.86 ± 0.2	4.84 ± 0.5	5.18 ± 0.0	0.69 ± 0.2	0.20 ± 0.0	5.0 ± 0.0
AB-3	5.18 ± 0.0	1.03 ± 0.0	1.03 ± 0.0	1.03 ± 0.0	0.53 ± 0.0	0.1 ± 0.0
AB-5	0.86 ± 0.2	0.86 ± 0.2	0.86 ± 0.2	0.86 ± 0.2	0.21 ± 0.0	5.0± 0.0
AB-6	0.69 ± 0.2	0.52 ± 0.0	0.69 ± 0.2	0.69 ± 0.2	0.53 ± 0.0	0.6 ± 0.0
AB-8	0.52 ± 0.0	0.52 ± 0.0	5.18 ± 0.0	0.69 ± 0.2	0.21 ± 0.0	6.7 ± 2.8
AB-9	0.52 ± 0.0	0.52 ± 0.0	4.49 ± 1.1	1.03 ± 0.0	0.53±0.0	5.0 ± 0.0
AB-10	5.18 ± 0.0	0.69 ± 0.2	5.18 ± 0.0	0.69 ± 0.2	0.53 ± 0.0	0.1 ± 0.0
AB-13	1.03 ± 0.0	0.52 ± 0.0	0.52 ± 0.0	1.03 ± 0.0	0.45 ± 0.0	1.3 ± 0.0
AB-15	1.03 ± 0.0	1.03 ± 0.0	5.18 ± 0.0	1.03 ± 0.0	5.3 ± 0.0	5.0 ± 0.0
AB-11Q	0.86 ± 0.2	1.03 ± 0.0	5.18 ± 0.0	1.03 ± 0.0	0.53 ± 0.0	0.1 ± 0.0
AB-14T	1.03 ± 0.0	4.66 ± 0.8	5.18 ± 0.0	5.18 ± 0.0	0.78 ± 0.2	0.1 ± 0.0
AB-16Q	1.03 ± 0.0	1.03 ± 0.0	5.18 ± 0.0	5.87 ± 1.1	0.88 ± 0.2	0.3 ± 0.0
AB-7T	4.49 ± 1.1	5.18 ± 0.0	5.18 ± 0.0	5.18 ± 0.0	0.53 ± 0.0	1.0 ± 0.30
ATCC 19606	0.69 ± 0.2	1.03 ± 0.0	0.86 ± 0.2	1.03 ± 0.0	0.47 ± 0.0	0.1 ± 0.0
Average MIC values (µg·mL^−1^)	1.65	1.60	3.38	1.78	0.79	2.70

(^a,b^) A statistically significant difference exists between the antimicrobial activity of cefepime and each of the tested E.Os and eugenol (Kruskal-Wallis test-Dunn’s multiple comparisons test, *p*-value < 0.0001).

**Table 2:** The determined MBC values of *Pimenta* spp. leaf and berry essential oils tested against *A. baumanii* isolate-8 (AB-8) and their MIC values equivalents.

Essential Oils/Standard Compound	MBC Value	MIC Values Equivalents
***P. dioica*** **leaf**	1.0 µg·mL^−1^	2× MIC
***P. racemosa*** **leaf**	2.0 µg·mL^−1^	4× MIC
***P. racemosa*** **berry**	2.76 µg·mL^−1^	4× MIC
***P. dioica*** berry *	More than 41.4 µg·mL^−1^	More than 8× MIC
**Eugenol**	1.2 µg·mL^−1^	6× MIC

* Concentration equivalent to 8× MIC caused only 2-log cycle reduction of the bacterial load.

**Table 3:** Relative percentile levels of volatiles identified in *Pimenta* berry and leaf essential oils using GC/MS.

RI * Calculated.	RI Reported	Name	Class	Essential Oil			
				*P. racemosa* Leaf	*P. dioica* Leaf	*P. racemosa* Berry	*P. dioica* Berry
**1186**	**1201**	**Decanal**	**aldehyde/ketone**	0.0	0.03	0.0	0.0
			**Total aldehyde/ketone**	0.0	0.03	0.0	0.0
906	932	α-Pinene		1.3	1.0	1.5	0.2
965.2	988	*β*-Myrcene		39.6	44.1	42.3	13.9
978.8	1003	*p*-Mentha-1(7),8-diene		0.9	0.2	2.9	0.0
980.9	1005	α-Phellandrene		1.0	0.6	0.6	1.0
991.2	1014	α-Terpinene		0.6	0.4	0.9	0.0
993	1014	4-carene		0.0	2.1	0.0	0.4
1001	1020	*p*-Cymene		2.3	2.1	0.9	0.4
1004.2	1024	Limonene		15.5	11.7	14.3	4.6
1019	1032	*β*-*cis*-Ocimene		2.8	0.6	4.6	1.1
1033	1054	*γ*-Terpinene		0.2	0.4	0.7	0.4
1063	1085	*p*-Mentha-2,4(8)-diene		0.1	0.0	0.4	0.3
			**Total Monoterpene hydrocarbons**	64.4	63.3	69.1	22.2
1009.5	1026	1,8-cineol	**Oxygenated monoterpene**	0.0	18.8	0.0	1.5
1080	1095	*β*-Linalool		2.1	5.3	1.6	3.6
1163	1174	Terpinen-4-ol		0.8	1.5	1.0	1.4
1181.5	1186	*α*-Terpineol		0.3	2.5	0.0	2.2
1250	1247	Chavicol		1.5	0.0	0.2	1.5
1250.1	1264	Geranial		0.0	0.0	0.0	0.6
			**Total oxygenated Monoterpene**	4.7	28.1	2.8	10.8
1334.5	1356	Eugenol	**Phenols**	31.0	8.6	27.7	65.6
1350	1345	α-Cubebene	**Sesquiterpene hydrocarbon**	0.0	0.0	0.0	0.1
1349	1374	α-Copaene		0.0	0.0	0.3	0.0
1396	1417	*β*-Caryophyllene		0.0	0.0	0.0	0.3
1430	1452	α-Humulene		0.0	0.0	0.0	0.2
1444.8	1484	Germacrene D		0.0	0.0	0.0	0.2
1478	1522	δ-Cadinene		0.0	0.0	0.0	0.6
			**Total sesquiterpenes**	0.0	0.0	0.3	1.4
			**Total**	100	100	100	100

RI *, retention index on DB-5-MS column relative to n-alkanes C8-C20.
